# Obstetrician and Gynecologist Physicians’ Practice Locations Before and After the *Dobbs* Decision

**DOI:** 10.1001/jamanetworkopen.2025.1608

**Published:** 2025-04-21

**Authors:** Becky Staiger, Valentin Bolotnyy, Sonya Borrero, Maya Rossin-Slater, Jessica Van Parys, Caitlin Myers

**Affiliations:** 1 Division of Health Policy and Management, University of California, Berkeley; 2 Hoover Institution, Stanford University, Stanford, California; 3 Department of Medicine, University of Pittsburgh, Pittsburgh, Pennsylvania; 4 Department of Health Policy, Stanford University, Stanford, California; 5 Department of Economics and Accounting, Hunter College, New York, New York; 6 Department of Economics, Middlebury College, Middlebury, Vermont

## Abstract

**Importance:**

State abortion policies may influence the practice locations of obstetricians and gynecologists (OBGYNs), having potentially significant implications for access to and quality of reproductive health care.

**Objective:**

To explore changes in OBGYN practice locations from before to after the *Dobbs v Jackson Women’s Health Organization* US Supreme Court decision in June 2022.

**Design, Setting, and Participants:**

National Plan & Provider Enumeration System data files were used in a descriptive cohort study assessing the association between state abortion policy environments and OBGYN practice locations in the US from January 1, 2018, to September 30, 2024, for all OBGYNs listed in the data files during the study period.

**Main Outcome and Measures:**

The number of OBGYNs practicing in states with differing abortion laws and the movement of OBGYNs between these states before and after the *Dobbs* decision.

**Results:**

The sample included 60 085 OBGYNs (59.7% women), of whom 3.8% were maternal-fetal medicine specialists and 12.9% were recent residency graduates. The mean increase in the per-quarter number of OBGYNs from before to after *Dobbs* was 8.3% (95% CI, 6.6%-10.1%) in states with total abortion bans, 10.5% (95% CI, 8.1%-13.0%) in states with gestational age limits or threatened bans, and 7.7% (95% CI, 5.9%-9.4%) in states with abortion protections. From the quarter immediately before *Dobbs* to the end of the study period, 95.8% of OBGYNs remained in protected states, 94.8% (95% CI, 94.3%-95.2%) remained in states threatening bans, and 94.2% (95% CI, 93.7%-94.7%) remained in states with abortion bans.

**Conclusions and Relevance:**

In this descriptive cohort study, there were no significant differences in trends in OBGYNs’ practice locations across states with different abortion-related policy environments after the *Dobbs* decision. Although these findings do not provide insight into changes in the quality of care provided, they suggest that there are no major changes in the supply of OBGYNs associated with the *Dobbs* decision.

## Key Points

**Question:**

How have practice locations of obstetricians and gynecologists (OBGYNs) changed since the *Dobbs v Jackson Women’s Health Organization* US Supreme Court decision in June 2022?

**Findings:**

In this cohort study of 60 085 OBGYNs, the number of OBGYNs did not significantly change across policy environments, increasing by 8.3% in states where abortion is banned, 10.5% in states where it is threatened, and 7.7% in states where it is protected after the *Dobbs* decision.

**Meaning:**

Although the *Dobbs* decision has increased physicians’ concerns about providing reproductive health care, there were no observed disproportionate changes in OBGYN practice location as of 2024.

## Teaser

This cohort study examines changes in obstetrician and gynecologist practice locations before vs after the *Dobbs v Jackson Women’s Health Organization* US Supreme Court decision.

## Introduction

In the 2 years since the US Supreme Court decision in *Dobbs v Jackson Women’s Health Organization* (2022) (*Dobbs*) overturned the constitutional right to abortion, 14 states have enforced bans on nearly all abortions, 6 states have enforced bans on abortions after 6 to 12 weeks’ gestation, and the future legality of abortion remains uncertain in several additional states where litigation and ballot initiatives are developing.^1,2,3^ Surveys of obstetricians and gynecologists (OBGYNs) have highlighted growing professional unease around increasing legal risks and constraints to practicing within established standards of care imposed by abortion bans.^4,5,6^ Numerous media reports have described physicians leaving states where abortion is banned in response to these concerns, including cases of retirement or migration out of Idaho,^7^ Florida,^8^ North Carolina,^8^ Ohio,^9^ Oklahoma,^10^ Tennessee,^11^ and Texas.^7,8^ However, besides these individual anecdotes, little is known about broader trends in OBGYNs’ practice locations since the *Dobbs* decision.

This descriptive cohort study used data from the National Plan and Provider Enumeration System (NPPES) to examine trends between January 2018 and September 2024 in the number of OBGYNs and share of all physicians who are OBGYNs practicing in the 14 states that implemented total abortion bans, the 11 states that implemented partial bans or where access is threatened, and the 25 states and the District of Columbia in which abortion care is protected. The study also examined the flow of OBGYNs between states with different policy environments from before to after the *Dobbs* decision.

## Methods

This cohort study was approved by the institutional review board of the University of California, Berkeley. A consent waiver was obtained via the institutional review board because the research involved no more than minimal risk of harm to the participants. This study followed the Strengthening the Reporting of Observational Studies in Epidemiology (STROBE) reporting guideline.

### Study Participants

Study participants were all practicing OBGYNs in the US, identified using the NPPES, a national registry of all health care practitioners in the US created and maintained by the US Centers for Medicare & Medicaid Services (CMS). Monthly NPPES data were compiled for the period of January 1, 2018, to September 30, 2024.^12,13^

The NPPES data contain National Provider Identifiers (NPIs), a unique 10-digit number assigned by the CMS to every health care practitioner, along with the practitioner’s name; gender; taxonomy codes (10-character alphanumeric codes that could be linked to a practitioner grouping [eg, allopathic and osteopathic physicians], a specialty [eg, obstetrics and gynecology], and a subspecialty [eg, maternal-fetal medicine])^14,15^; business and practice location addresses (including state); state medical license number; and the NPI deactivation date, if applicable. A physician’s NPI may be deactivated due to retirement, death, or fraudulent use.^16^ The NPPES does not report data on race.

To identify physicians’ year of graduation from medical school, the NPPES data were supplemented by IQVIA OneKey data and the CMS Doctors and Clinicians national downloadable file.^17^ IQVIA OneKey is a nonpublic database that contains a wealth of clinician information, some of which is not available in the NPPES (such as graduation year). The CMS Doctors and Clinicians data are publicly available and were used to fill graduation years missing in the IQVIA data, when possible.

The primary population of interest was allopathic or osteopathic attending physicians or trainees (including resident physicians) with a nonmissing practice or business state and a primary or secondary taxonomy associated with obstetrics and gynecology who were observed in the data at any point between January 1, 2018, and September 30, 2024. There were 3 populations of interest that were considered in secondary analyses. First, OBGYNs who graduated from their medical residency program in the prior academic year were examined because many physicians relocate for new professional opportunities after residency graduation. OBGYN residency graduates between January 1, 2018, and September 30, 2023, were identified using their year of graduation from medical school; additional details on this approach are described in the eMethods in Supplement 1. Second, maternal-fetal medicine (MFM) specialists were examined because abortion restrictions may be particularly salient to physicians who focus on care for complex pregnancies. Complex family planning specialists, although also uniquely impacted by abortion bans, are too new a subspecialty to allow for pre-post *Dobbs* comparisons. Third, OBGYNs were analyzed separately based on reported gender (men or women) in the NPPES database. Gender may be a relevant factor if OBGYNs consider practice locations that are associated with their own needs for access to reproductive health care.

### Exposure

**Figure 1.**
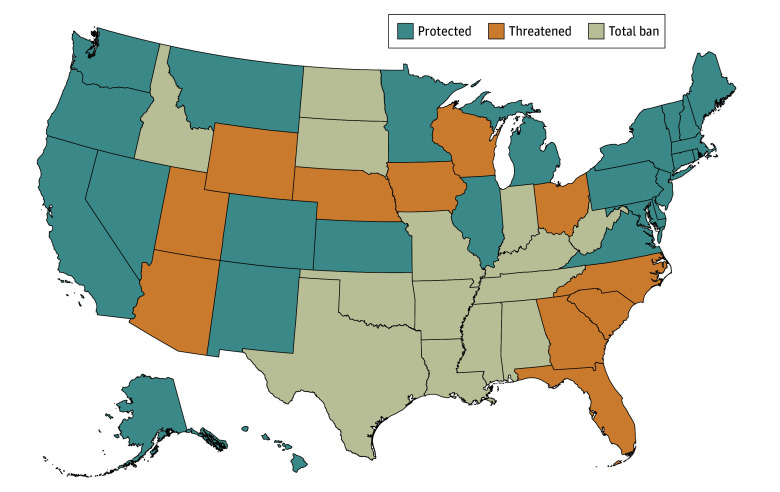
Abortion Ban Status as of August 2024 The 14 total ban states are Alabama, Arkansas, Idaho, Indiana, Kentucky, Louisiana, Mississippi, Missouri, North Dakota, Oklahoma, South Dakota, Tennessee, Texas, and West Virginia. The 11 threatened states are Arizona, Florida, Georgia, Iowa, North Carolina, Nebraska, Ohio, South Carolina, Utah, Wisconsin, and Wyoming. The 25 protected states are Alaska, California, Colorado, Connecticut, Delaware, Hawaii, Illinois, Kansas, Massachusetts, Maryland, Maine, Michigan, Minnesota, Montana, New Hampshire, New Jersey, New Mexico, Nevada, New York, Oregon, Pennsylvania, Rhode Island, Virginia, Vermont, and Washington, plus the District of Columbia.

States’ abortion policies were categorized according to *The New York Times’* ongoing tracking of the legal landscape^1^ and are mapped in Figure 1. Each state was assigned to 1 of 3 categories based on their policy environments in August 2024: (1) total ban states enforced bans on all or most abortions (14 states); (2) threatened states enforced gestational age bans and/or were classified as likely to enforce a gestational-age or total ban in the future (11 states); and (3) protected states that did not enforce an abortion ban and were not considered likely to do so (25 states and the District of Columbia).

### Outcomes

There were 2 main outcomes of interest. The first was the count of OBGYNs per quarter who were observed practicing in total ban, threatened, and protected state policy environments. The second was the share of all physicians who were OBGYNs in each quarter by practice environment. Secondary outcomes included the share of OBGYNs who had recently graduated from residency who were observed practicing in a particular policy environment and who had trained in either the same (concordant) or different (discordant) policy environment. Additionally, the percentage change in OBGYN count relative to 2022 quarter (Q) 1 was evaluated.

### Secondary Analyses

The share of recently graduated OBGYN residents in a given policy environment in the first academic year (July to June) after their residency was calculated as the number of residency graduates observed practicing in that policy environment divided by all other residents in their graduation-year cohort. This was further stratified by the policy environment of the state in which they did their residency training. Unadjusted trends (as in the main analysis) in the number and share of MFM specialists and male and female OBGYNs during the study period were also examined.

The percentage change in OBGYN counts was defined in each quarter relative to OBGYN counts in the quarter before the *Dobbs* decision (2022 Q1). To contextualize trends of OBGYN location within broader trends in physician practice location, the percentage change of OBGYN counts was analyzed relative to the percentage change of pediatrician, anesthesiologist, and internist counts for total ban states. These specialties were consistently the most common among physicians in the sample and less likely to be directly impacted by abortion bans than other specialties (such as family medicine practitioners).

### Sensitivity Analysis

To test the sensitivity of the results to alternative measures of location, 2 additional analyses were run. First, the percentage change (relative to 2022 Q1) in the number of medical licenses per quarter associated with a given policy environment, as well as the share of OBGYNs with multiple state licenses, was explored during the study period. OBGYNs with multiple state licenses included OBGYNs practicing simultaneously in multiple states or OBGYNs anticipating a move to (or having just moved from) another state. Second, unadjusted trends in OBGYN location using business (as opposed to practice) addresses were examined during the study period. Additional details on the difference between practice and business locations are given in the eMethods in Supplement 1.

### Statistical Analysis

The main analysis calculated the total OBGYN count and share in each policy environment × quarter cell (ie, 3 policy environments × 27 quarters from 2018 Q1 to 2024 Q3 = 81 observations) to document unadjusted trends in OBGYNs’ practice locations around the *Dobbs* decision. The denominator in the OBGYN share was the total number of physicians of any specialty (per their primary or secondary taxonomy).

To compare mean changes in the count and share of OBGYNs across policy environments in the quarters before and after the *Dobbs* decision, we used Poisson models regressing each outcome on dichotomous variables equal to 1 in the post–*Dobbs* quarters (2022 Q2 to 2024 Q3) and 0 otherwise (2018 Q1 to 2022 Q1) for each ban category. Postestimation pairwise *t* tests were performed to examine whether the estimated changes were different in the 3 policy environments. 95% CIs were obtained from 2-tailed *t* tests.

The movement of OBGYNs among protected, threatened, and total ban state groups from the quarter before the *Dobbs* decision (2022 Q1) into the last quarter of the data (2024 Q3) was visualized in a flow diagram. To account for potential responses that include entering or exiting practice all together, we also included OBGYNs who were not observed practicing in 2022 Q1, ie, who were not yet practicing as an OBGYN or who had not yet registered their NPI, possibly due to being in training; and OBGYNs who were no longer observed practicing in 2024 Q3, ie, exiters who may have retired, changed their specialty from OBGYN to a different specialty, died, or lost their license due to a disciplinary action. Except for a change in specialty, the reason for exit was unobservable in the data.

To determine whether the probability that an OBGYN remained in or exited from a policy environment differed by policy environment, we used linear probability models with individual OBGYN-level data in which the outcome indicated whether an individual OBGYN remained in or exited the policy environment. The explanatory variable was a dichotomous variable indicating the policy environment of the OBGYN’s practice state in the quarter before the *Dobbs* decision (2022 Q1). A 2-sided *P* < .05 was considered statistically significant. We clustered SEs at the physician level. All data analysis was performed using Stata MP software, version 18.5 (StataCorp LLC).

## Results

**Figure 2.**
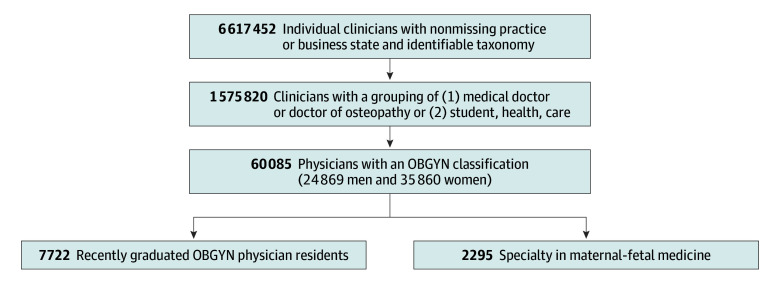
Number of Clinicians by Each Restriction Step and in Each Subsample Summing the number of men and women may generate totals greater than the reported total due to inconsistently recorded gender. OBGYN indicates obstetrician-gynecologist.

Figure 2 presents a flowchart of the construction of the final analytical sample. From all practitioners and organizations in the raw NPPES files, the sample was first restricted to 6 617 452 individual practitioners. Of these, 1 575 820 were identified as physicians or trainees of any specialty, 60 085 of whom were OBGYNs (59.7% women and 40.3% men). Of these OBGYNs, 12.9% were recent residency graduates and 3.8% were MFM specialists.

**Figure 3.**
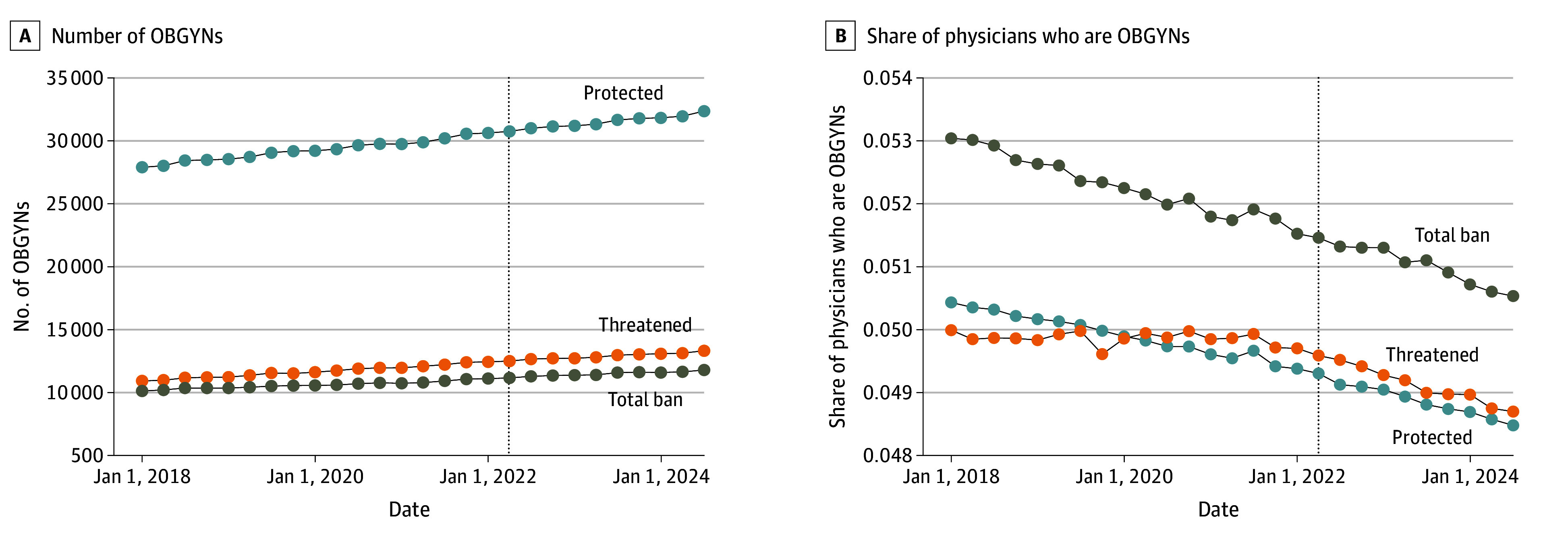
Unadjusted Trends in the Number of Obstetricians-Gynecologists (OBGYNs) in Protected, Threatened, and Total Ban States Trends are unadjusted and calculated at the policy environment level. Dashed vertical lines indicate the quarter in which the *Dobbs* decision was released (2022 quarter 2).

Figure 3 presents trends in OBGYN counts and shares during the study period (2018 Q1 to 2024 Q3). The trends in both counts and shares were similar across state policy environments. Poisson regressions estimated increases in mean per-quarter counts of 8.3% (95% CI, 6.6%-10.1%) in total ban states, 10.5% (95% CI, 8.1%-13.0%) in threatened states, and 7.7% (95% CI, 5.9%-9.4%) in protected states. The differences in these increases were not statistically significant for total ban vs protected environments or for threatened vs protected environments. Similarly, Poisson regressions estimated decreases in shares of OBGYNs of 2.4% (95% CI, 1.8%-2.9%) in total ban states, 1.5% (95% CI, 1.1%-1.8%) in threatened states, and 2.1% (95% CI, 1.6%-2.5%) in protected states. The differences in these decreases were not statistically significant for total ban vs protected environments, although they were statistically significant for threatened vs protected environments (*P* = .045).

**Figure 4.**
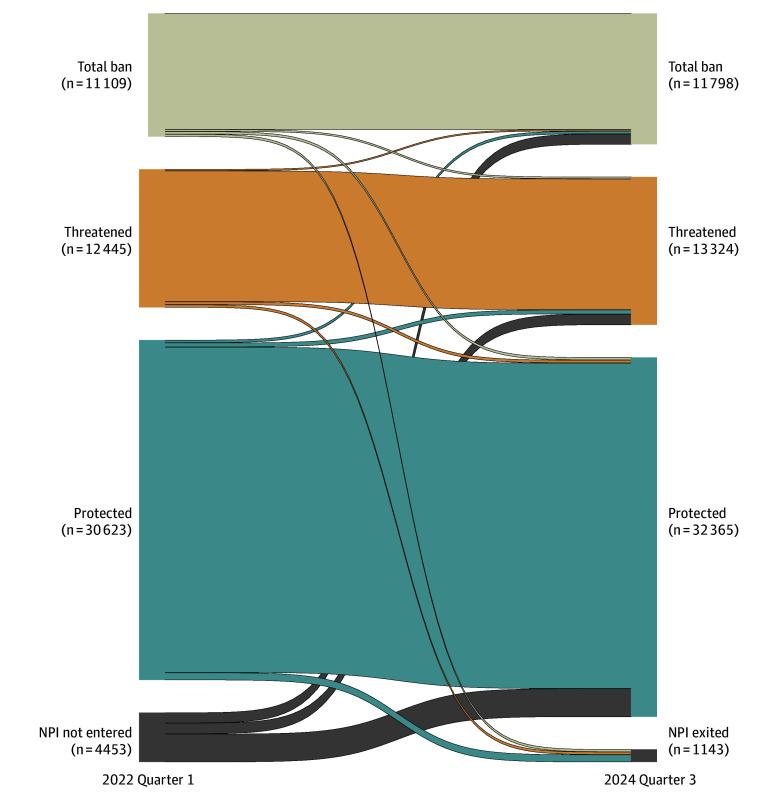
OBGYN Practice Location Movement Among Policy Environments, 2022 Quarter 1 to 2024 Quarter 3 NPI indicates National Provider Identifier.

Figure 4 provides a flow diagram illustrating changes in OBGYN status and location between 2022 Q1, the quarter before the *Dobbs* decision, and 2024 Q3, the last quarter in the study period. There were no meaningful differences in the movement of OBGYNs among different policy environments. A total of 95.8% of OBGYNs in protected states remained in protected states compared with 94.8% (95% CI, 94.3%-95.2%) and 94.2% (95% CI, 93.7%-94.7%) of OBGYNs in threatened and total ban states, respectively. In protected states, 2.2% of OBGYNs exited the data entirely, compared with 2.0% (95% CI, 1.7%-2.3%) and 2.1% (95% CI, 1.8%-2.4%) of OBGYNs in threatened and total ban states, respectively.

### Secondary Analyses

**Figure 5.**
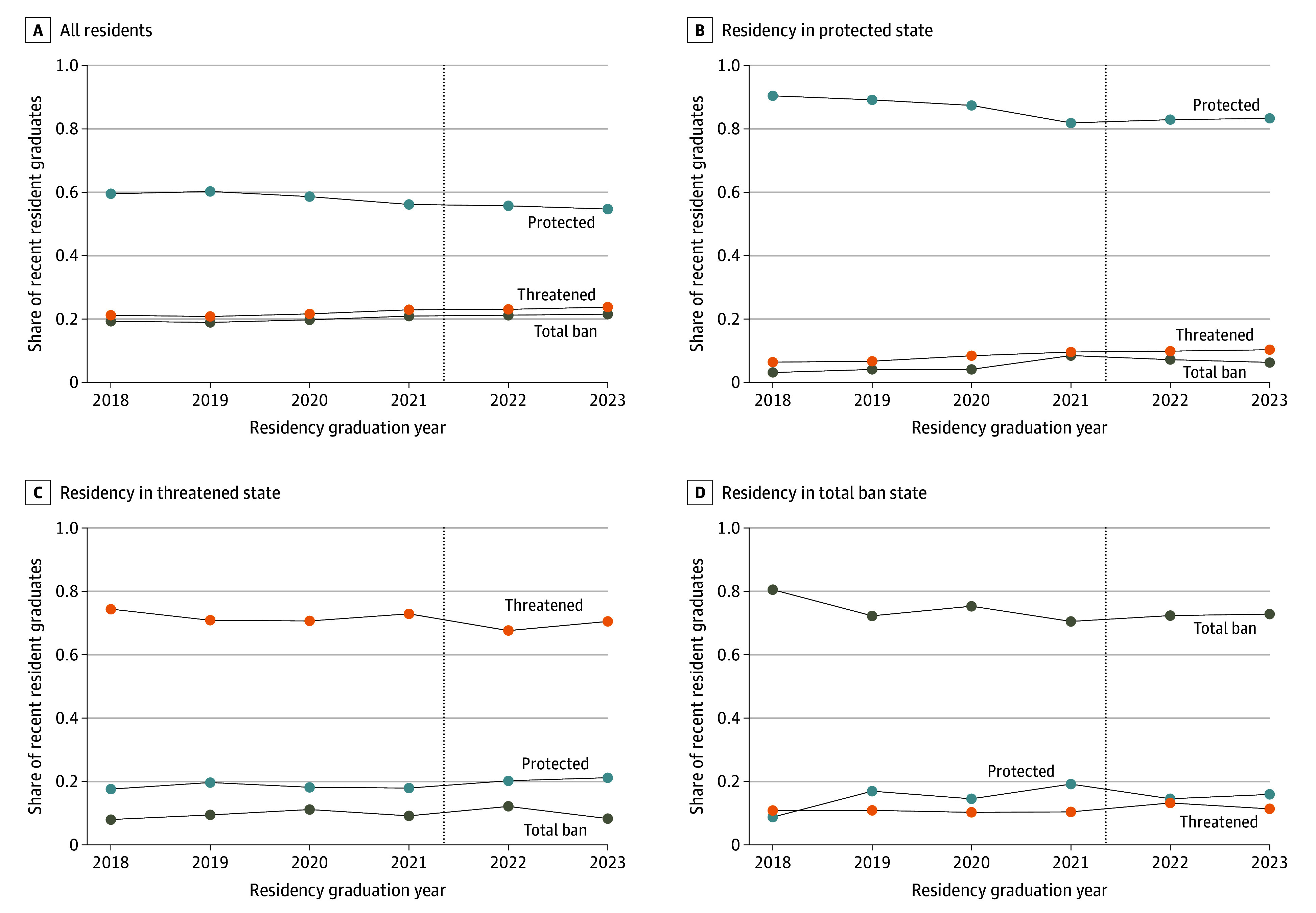
Postresidency Policy Environment by Residency Graduation Cohort Dashed vertical lines indicate the quarter in which the *Dobbs* decision was released (2022 quarter 2).

Trends in the policy environment of postresidency location among recent residency graduates were relatively stable over time, including around the *Dobbs* decision (Figure 5A). Concordant cases were much more common than discordant cases (Figure 5B-D). The share of recent graduates in protected states increased by less than 2 percentage points after the *Dobbs* decision, whereas the share of recent graduates in total ban states decreased by less than 4 percentage points after the *Dobbs* decision.

Among other secondary populations of interest, no discernible difference in practice location was observed. On average, trends in the practice locations of MFMs evolved similarly from 2018 Q1 to 2024 Q3 across policy environments (eFigure 1 in Supplement 1), and flow across policy environments between 2022 Q1 and 2024 Q3 was similar as well (eTable 1 in Supplement 1). The count of OBGYNs who were women and the share of all physicians who were OBGYNs and women increased similarly across all policy environments (eFigure 2 in Supplement 1). The count of OBGYNs who were men was relatively flat for all policy environments during the study period, and the share of all physicians who were OBGYNs and men decreased during the period for all policy environments (eFigure 3 in Supplement 1).

Across total ban states, state-specific trends in OBGYN growth compared with 2022 Q1 were comparable to trends in other specialties (eFigure 4 in Supplement 1). eTable 2 in Supplement 1 reports changes in OBGYN counts from the quarter before the *Dobbs* decision (2022 Q1) to 2024 Q3 across all states.

### Sensitivity Analysis

Increases in the number of state medical licenses were comparable across the 3 policy environments (eFigure 5A in Supplement 1). Trends in the share of OBGYNs with more than 1 state license were similar across policy environments as well (eFigure 5B in Supplement 1). Trends in the count and share of OBGYNs using business location were qualitatively similar to trends using practice location (eFigure 6 in Supplement 1).

## Discussion

In this descriptive cohort study examining the practice locations of OBGYNs before and after the *Dobbs* decision, trends in the number of OBGYNs in states where abortion is protected, threatened, or banned were similar. The only statistically significant difference suggested that the share of physicians who are OBGYNs decreased less in threatened states than in protected ones, opposite to the expected finding if OBGYNs were leaving states where abortion is threatened. Moreover, there were no appreciable differences among recent OBGYN residency graduates in the likelihood of moving to total ban states. Discernible differences by OBGYN subspecialty and gender were also not observed.

These results may suggest that concerns prompting OBGYNs to consider relocation are thus far being offset by other forces, such as ties to patients and local community and the significant effort and disruption associated with relocation.^5^ Such findings are also consistent with recent work showing little to no difference in the post-*Dobbs* change in per-capita OBGYN entrance into states with and without abortion bans.^18^

This study did not find any meaningful difference in the share of residents who move into protected, threatened, or total ban states before and after the *Dobbs* decision. Recent, related studies documented a decrease in graduating US medical school students applying for residencies in states with abortion bans compared with other states.^19,20^ However, all residency positions were ultimately filled, suggesting that although application numbers might be an indicator of OBGYN preferences, they do not reflect a net effect on the number of practicing residents in ban states. Future work should consider whether the types of residents matching to ban states and their satisfaction with their training may be affected by bans.

Fewer than 20% of office-based OBGYNs report providing abortion services for unwanted pregnancies,^4^ yet termination may also be required to treat complications of pregnancy, such as placental abruption, placenta previa, preeclampsia, eclampsia, and ectopic pregnancies.^21^ Survey and anecdotal evidence suggests that the *Dobbs* ruling and subsequent abortion bans have forced OBGYNs to provide care that is different from established standards, compromising patients’ health and well-being^22^ and in some cases resulting in maternal deaths.^23^ A number of OBGYNs report feeling moral distress.^5,22^ In one recent qualitative study of 54 OBGYNs practicing in states that banned abortion in response to the *Dobbs* decision, clinicians reported feeling “muzzled, handcuffed, or straitjacketed”^5^ by legal restrictions on abortion services.

Although the analyses presented here suggest that trends in the number of OBGYNs practicing in states with abortion bans do not meaningfully differ from trends in abortion-protecting states after the *Dobbs* decision, they do not speak to the evolution of other aspects of reproductive health care, including the quality of care being provided to patients, the moral distress felt by practitioners, and the quality of training being provided to OBGYN residents. These analyses also do not comprehensively address how the broader landscape of reproductive health care provision has changed, including the delivery of abortion medication across state lines and the expansion of telehealth. Future work should focus on the impact of *Dobbs* on these important considerations as well as on the continuing evolution of OBGYN practice locations.

### Limitations

This study has several limitations. First, our analysis focuses on the 2 years after the *Dobbs* decision, a time frame that may be more likely to capture short-term changes in physician behavior, such as adaptation of practices to new regulatory environments, vs longer-term responses, such as moving from a particular policy environment. Second, physician characteristics (such as political party affiliation) that may be correlated with a physician’s choice to move to (and remain in) states with particular policy environments are not observable. Future work should explore this “sorting” explanation in more detail. Third, the NPPES dataset has only been verified for physician location accuracy for physicians who are billing public and private insurers, although this likely captures most physicians.^24,25^ Moreover, the NPPES has been found to include more accurate physician location information than 2 other widely used databases (SK&A Healthcare Data and the American Medical Association Masterfile).^26,27^ Fourth, it is likely that some measurement error in physician location exists; exercises to document movement of physicians named in media reports confirmed an error rate of approximately 20%, which is in line with prior work.^26^ There is, however, no indication that this error rate would bias the results reported here in any particular direction. For the error rate of physician practice location to bias our results in a qualitatively meaningful way, the error would have to be disproportionately from OBGYNs who move out of total ban states to protected or threatened states after the *Dobbs* decision. We have no reason to think that those OBGYNs would be less likely to update their practice location than other OBGYNs. Additional details are provided in the eMethods in Supplement 1. Fifth, the analysis does not describe the practice locations of all individuals who provide different forms of reproductive health care, such as midwives or family planning physicians without a clearly stated OBGYN specialty, and others. We leave this for future work. Sixth, the trends focused on in this study cannot be characterized as causal. In this empirical setting, all physicians (OBGYNs and other specialists in ban, threatened, and protected states) are essentially in the treatment group in the sense that any state implementing an abortion ban may impact their likelihood of moving to that state (or moving, in general) for personal and professional reasons. The lack of a well-defined control group complicates the application of quasi-experimental methods, such as difference-in-differences, thus making causal inference difficult.

## Conclusions

This descriptive cohort study used administrative records on physician practice locations to document that trends in OBGYN counts in states where abortion is banned, threatened, or protected have evolved similarly around the US Supreme Court’s *Dobbs* decision. The percentage of OBGYNs switching policy environments was between 1% and 2% across all 3 policy environments. The study was not able to observe the full extent to which the quality of training, care, and outcomes in reproductive health care have changed across states since the *Dobbs* decision. Although these findings do not provide insight into changes in the quality of care provided, they suggest that there are no major changes in the supply of OBGYNs associated with the *Dobbs* decision. Future research should assess changes in quality of care as well as whether OBGYN practice location patterns change in the long run.

## Notes

Supplement 1. **eMethods.** Supplemental Methods **eTable 1.** Maternal-Fetal Medicine (MFM) Specialist Movement Across Policy Environments, 2022Q1–2024Q3 **eTable 2.** OBGYN Counts by State, 2022Q1–2024Q3 **eFigure 1.** Trends in the Count of Maternal-Fetal Medicine (MFM) Specialists per Policy Environment **eFigure 2.** Trends in the Count of OBGYNs Who Are Women per Policy Environment **eFigure 3.** Trends in the Count of OBGYNs Who Are Men per Policy Environment **eFigure 4.** Trends in the Percent Change in OBGYNs, Pediatricians, Internists, and Anesthesiologists in Total Ban States **eFigure 5.** Trends in State Licenses Associated With OBGYNs, 2018–2024 **eFigure 6.** Trends in the Count of OBGYNs per Policy Environment, Business State Definition

Supplement 2.
Data Sharing Statement
