# Supplementary material for: Obstetrician and Gynecologist Physicians’ Practice Locations Before and After the Dobbs Decision
Source: JAMA Netw Open. 2025 Apr 21;8(4):e251608. doi: 10.1001/jamanetworkopen.2025.1608 (PMC12013358; doi:10.1001/jamanetworkopen.2025.1608)
Supplement: Supplement 1. — eMethods. Supplemental Methods eTable 1. Maternal-Fetal Medicine (MFM) Specialist Movement Across Policy Environments, 2022Q1–2024Q3 eTable 2. OBGYN Counts by State, 2022Q1–2024Q3 eFigure 1. Trends in the Count of Maternal-Fetal Medicine (MFM) Specialists per Policy Environment eFigure 2. Trends in the Count of OBGYNs Who Are Women per Policy Environment eFigure 3. Trends in the Count of OBGYNs Who Are Men per Policy Environment eFigure 4. Trends in the Percent Change in OBGYNs, Pediatricians, Internists, and Anesthesiologists in Total Ban States eFigure 5. Trends in State Licenses Associated With OBGYNs, 2018–2024 eFigure 6. Trends in the Count of OBGYNs per Policy Environment, Business State Definition [file jamanetwopen-e251608-s001.pdf]

Supplemental Online Content

Staiger B, Bolotnyy V, Borrero S, Rossin-Slater M, Van Parys J, Myers C. Obstetrician and gynecologist physicians' practice locations before and after the *Dobbs* decision. *JAMA Netw Open*. 2025;8(4):e251608. doi:10.1001/jamanetworkopen.2025.1608

eMethods. Supplemental Methods

eTable 1. Maternal-Fetal Medicine (MFM) Specialist Movement Across Policy Environments, 2022Q1–2024Q3

eTable 2. OBGYN Counts by State, 2022Q1–2024Q3

eFigure 1. Trends in the Count of Maternal-Fetal Medicine (MFM) Specialists per Policy Environment

eFigure *2*. Trends in the Count of OBGYNs Who Are Women per Policy Environment

eFigure 3. Trends in the Count of OBGYNs Who Are Men per Policy Environment

eFigure 4. Trends in the Percent Change in OBGYNs, Pediatricians, Internists, and Anesthesiologists in Total Ban States

eFigure 5. Trends in State Licenses Associated With OBGYNs, 2018 -- 2024

eFigure 6. Trends in the Count of OBGYNs per Policy Environment, Business State Definition

This supplemental material has been provided by the authors to give readers additional information about their work.

eMethods. Supplemental Methods

Identifying OBGYN Residents
To identify OBGYN residents, we take the following steps:

1. Create the set of providers who are ever classified as OBGYNs, but may be classified as another taxonomy (e.g., student) in some quarters.
2. Use the year of graduation to identify residents. We define residents (and year of residency) based on academic year from medical school graduation. The table immediately below illustrates residency year classifications for someone who graduated in 2016:

Calendar Year-Months	Residency Year
July 2016 - June 2017	R1
July 2017 - June 2018	R2
July 2018 - June 2019	R3
July 2019 - June 2020	R4
After July 2020	Graduate

Notably, there is a taxonomy code in the NPPES data that indicates that the provider is a "Student in an Organized Health Care Education/Training Program" (390200000X). However, there are two issues with an approach that would flag residents based on the presence of taxonomy codes:

- Often, an NPI will have a "student" taxonomy code, but no additional detail on what type of physician the student is (e.g. OBGYN or other).
- Sometimes, a physician will retain their "student'" taxonomy code for several quarters/years after finishing their residency.

Our analysis focuses on recent residency graduates for whom we observe their R4 year. Because of this condition, we drop residents who do not have an identifiable state of residency (due to a lack of sufficient post-period in order to identify OBGYN residents). The table immediately below reports the number of OBGYNs removed from the sample after imposing this restriction.

Year of Residency Graduation*	No Restriction	Must Observe Residency Information
2018	1230	1223
2019	1242	1230
2020	1181	1175
2021	1217	1209
2022	1145	1141
2023	992	989
2024	757	755

*Year of residency graduation is calculated as (known) year of graduation from medical school plus four years, representing the standard four years of OBGYN residency.

For reference, the table immediately below reports counts of residents (by year in residency and calendar cohort year) reported by the American Council For Graduate Medical Education (ACGME) in the "Data Resource Book." Notably, while our analysis almost certainly

undercounts the total number of R4 OBGYNs due to the nature of the data, it is not clear how our definition of OBGYN differs from that used by ACGME.

Year Cohort	R1	R2	R3	R4	Total
2019-2020	1460	1427	1408	1382	5677
2020-2021	1499	1448	1416	1406	5769
2021-2022	1517	1479	1441	1424	5861
2022-2023	1560	1496	1474	1432	5962

Practice vs. Business State

The following definitions are based on the instructions given to providers when they submit their information to the NPPES.[1] A provider's practice location is defined as the "location where the actual services are rendered." Their business location is included so that the NPPES/CMS may "contact [the provider] directly to resolve any issues that may arise during our review of [their] application" and can be any address where they receive mail.

A provider's (non-missing) practice state can differ from their (non-missing) business state. For all providers in our final sample (including non-OBGYNs), 93% of business and practice states match. Average match rates are similar across protected (94.0%), threatened (93.8%), and total ban (93.3%) policy environments.

NPPES Location Accuracy

Under federal law, every healthcare provider in the United States that is covered by the Health Insurance Portability and Accountability Act (HIPAA) is required to apply for a unique National Provider Identifier (NPI), which is issued by the Centers for Medicare and Medicaid Services (CMS) through the National Plan and Provider Enumeration System (NPPES). Specifically, any healthcare provider who transmits health information on behalf of themselves or their business must have an NPI to be HIPAA compliant.[1] Generally, providers are assigned an NPI upon starting a residency program, largely to facilitate their prescription of pharmaceuticals to patients.[2] In practice, for OBGYNs with a non-missing graduation year and who graduated within 4 years of our first year of data (i.e., after 2014) we observe that approximately 99% appear in the NPPES database at least once during their residency. Notably, only about 60% appear in all four years of residency. This is likely due to the fact that residents submit bills for services delivered using their attending physicians' NPI, and thus for at least a subset of providers, having an NPI is not necessary prior to residency graduation.[3]

The accuracy and up-to-date nature of the NPPES data are of paramount importance for an analysis of physician migration. There are several reasons we believe these data to be the best available for the purposes of our analyses. First, one provision of Section 162.410 of the 2004 HHS Final Rule[1] is a requirement that physicians update changes to the required NPI data fields within 30 days of that change occurring. Those required fields include the physician's specialty, mailing address, and business/practice address, among other fields. Failure to comply would technically be a violation of federal law. Second, insurance companies use the NPPES data to verify the veracity of submitted claims, with incorrect address information potentially leading to

---

[1] https://nppes.cms.hhs.gov/webhelp/nppeshelp/ADDRESS%20PAGE.html

© 2025 Staiger B et al. *JAMA Network Open*.

canceled or delayed claim processing.[4,5] Section 162.410 of the 2004 HHS Final Rule stipulates that the NPI be used by covered entities for all standard transactions, making it important for physicians to maintain accurate underlying information in order to be paid for the services they provide.

Third, previous academic work has confirmed the accuracy of physicians' contact information found in the NPPES. Authors in one study performed random audits of these data by calling the phone numbers on file and found that 86% of physicians in the data had accurate mailing address information.[6] In contrast, they found that 85% of physicians in the SK&A Healthcare Data and 42% of physicians in the American Medical Association Masterfile, alternative datasets for identifying physician locations, had accurate mailing addresses. The study focused on several specialties which unfortunately did not include Gynecology or Obstetrics. However, for Family Medicine, the NPPES had accurate address information 88% of the time (92% for SK&A and 54% for the AMA Masterfile) and for Internal Medicine the NPPES was accurate 94% of the time (79% for SK&A and 37% for the AMA Masterfile). Though not perfect, the high address accuracy rate found in the NPPES by this study is reassuring, as is the fact that the NPPES appears to outperform in address accuracy relative to other prominent physician databases.

Fourth, information provided by the American College of Obstetricians and Gynecologists (ACOG) and Doximity, a professional network for physicians, reassure us that the data we are using are capturing most of the actively practicing OBGYNs. A 2017 ACOG report[7] described a membership of 35,586 Fellows, while a Doximity analysis of OBGYN shortages using its own data worked with 2017 practice location data of more than 43,000 OBGYNs.[8] The data we work with had 51,146 OBGYNs in 2018, in the ballpark of these other analyses.

Finally, as an additional check on the accuracy of the NPPES database, a list of OBGYN providers who had been reported in news articles as having moved out of total ban states after the Dobbs decision was compiled. Provider names were linked to NPIs using web searches, and NPIs were subsequently searched for in the NPPES database to determine whether provider location and evidence of migration matched what had been reported in the news articles. Of the ten providers identified, we were able to observe the relocations corresponding to the news article for seven using practice state (six were exactly correct, and one was partially correct) and for nine using business state (five were exactly correct, and four were partially correct). Though this validation was performed on a small sample, the results were in line with the general accuracy of the NPPES address data as described above and motivated us to confirm the robustness of our analysis of OBGYN migration patterns using both the practice state and the business state information in our data.

eReferences

1. Department of Health and Human Services. 45 CFR Part 162: HIPAA Administrative Simplification: Standard Unique Health Identifier for Health Care Providers; Final Rule. *Federal Register*. 2004;69(15). https://www.cms.gov/regulations-and-guidance/administrative-simplification/nationalprovidentstand/downloads/npifinalrule.pdf

2. Do Medical Students, Interns, and Residents Need National Provider Identifiers? AAMC. Accessed October 17, 2024. https://www.aamc.org/professional-development/affinity-groups/gir/viewpoint-provider-identifiers

© 2025 Staiger B et al. *JAMA Network Open*.

3. Understanding the National Provider Identifier. Accessed October 17, 2024. https://www1.deltadentalins.com/about/legal/understanding-npi.html

4. Update on claims processing - Provider News. Accessed October 17, 2024. https://providernews.anthem.com/maine/articles/update-on-claims-processing-9265

5. New strategic provider system implementing August 2023 - Provider News. Accessed October 17, 2024. https://providernews.anthem.com/nevada/articles/new-strategic-provider-system-implementing-august-2023-13011

6. DesRoches CM, Barrett KA, Harvey BE, et al. The Results Are Only as Good as the Sample: Assessing Three National Physician Sampling Frames. *J Gen Intern Med*. 2015;30(Suppl 3):595-601. doi:10.1007/s11606-015-3380-9

7. Rayburn WF. *The Obstetrician-Gynecologist Workforce in the United States: Facts, Figures, and Implications, 2017*. American Congress of Obstetricians and Gynecologists; 2017.

8. Doximity. *2018 OB-GYN Workforce Study. Looming Physician Shortages: A Growing Women's Health Crisis*.; 2018. https://press.doximity.com/reports/ob-gyn-workload-and-potential-shortages-2018.pdf

© 2025 Staiger B et al. *JAMA Network Open*.

eTable 1. Maternal-Fetal Medicine (MFM) Specialist Movement Across Policy Environments, 2022Q1 – 2024Q3

2022Q1	Protected (% Change)	Threatened (% Change)	Total Ban (% Change)	Exit (% Change)	Total
Out	61 (61)	14 (14)	25 (25)		100
Protected	1,115 (95)	19 (2)	21 (2)	13 (1)	1,168
Threatened	16 (4)	422 (93)	12 (3)	4 (1)	454
Total Ban	13 (3)	11 (3)	392 (93)	7 (1)	423
Total	1,205	466	450	24	

Notes: Percent change is calculated relative to the row total.

© 2025 Staiger B et al. *JAMA Network Open*.

eTable 2. OBGYN Counts by State, 2022Q1 – 2024Q3

State	Policy Environment	2022Q1	2024Q3	State	Policy Environment	2022Q1	2024Q3
AK	Protected	129	135	MT	Protected	168	170
AL	Total Ban	627	674	NC	Threatened	1,759	1,884
AR	Total Ban	342	354	ND	Total Ban	101	102
AZ	Threatened	972	1,025	NE	Threatened	311	328
CA	Protected	6,028	6,365	NH	Protected	252	283
CO	Protected	1,025	1,104	NJ	Protected	1,664	1,729
CT	Protected	855	919	NM	Protected	323	320
DC	Protected	290	323	NV	Protected	397	442
DE	Protected	174	188	NY	Protected	4,155	4,321
FL	Threatened	3,174	3,443	OH	Threatened	1,931	2,033
GA	Threatened	1,670	1,810	OK	Total Ban	505	546
HI	Protected	320	336	OR	Protected	764	832
IA	Threatened	393	419	PA	Protected	2,523	2,596
ID	Total Ban	210	227	RI	Protected	262	274
IL	Protected	2,406	2,512	SC	Threatened	839	882
IN	Total Ban	1,015	1,078	SD	Total Ban	112	116
KS	Protected	390	429	TN	Total Ban	986	1,057
KY	Total Ban	679	702	TX	Total Ban	4,119	4,450
LA	Total Ban	739	775	UT	Threatened	442	486
MA	Protected	1,444	1,487	VA	Protected	1,381	1,524
MD	Protected	1,245	1,342	VT	Protected	117	120
ME	Protected	207	225	WA	Protected	1,199	1,288
MI	Protected	1,957	2,077	WI	Threatened	873	930
MN	Protected	947	1,023	WV	Total Ban	232	236
MO	Total Ban	1,078	1,103	WY	Threatened	80	83
MS	Total Ban	364	377				

Notes: "State" is the OBGYN's practice state in a given year-quarter.

eFigure 1. Trends in the Count of Maternal-Fetal Medicine (MFM) Specialists per Policy Environment

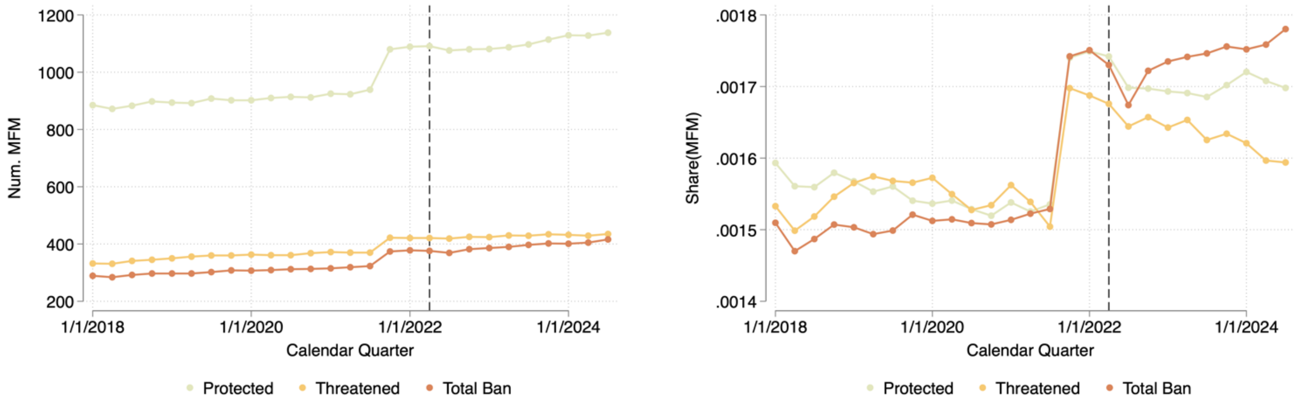

(a) Count of MFM Specialists

(b) Share of all physicians who are MFM Specialists

© 2025 Staiger B et al. *JAMA Network Open*.

eFigure 2. Trends in the Count of OBGYNs Who Are Women per Policy Environment

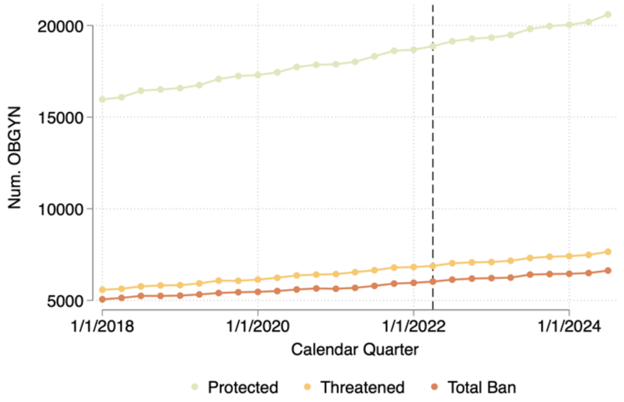

(a) Count of OBGYNs who are women

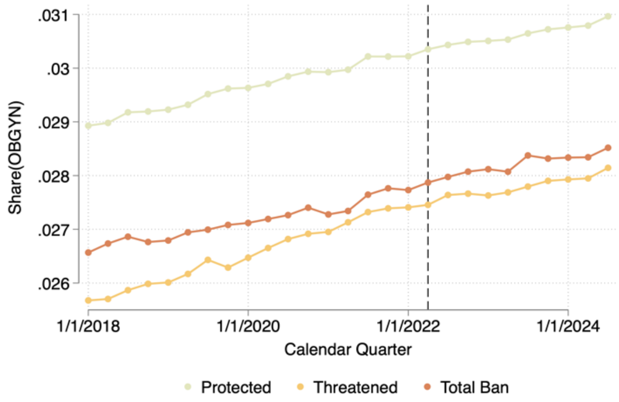

(b) Share of all physicians who are OBGYNs and women

© 2025 Staiger B et al. *JAMA Network Open*.

eFigure 3. Trends in the Count of OBGYNs Who Are Men per Policy Environment

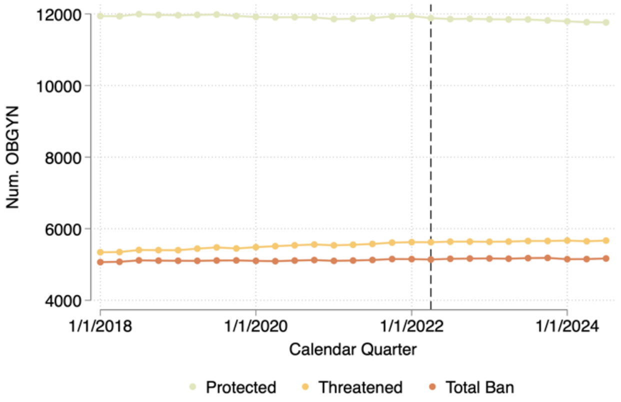

(a) Count of OBGYNs who are men

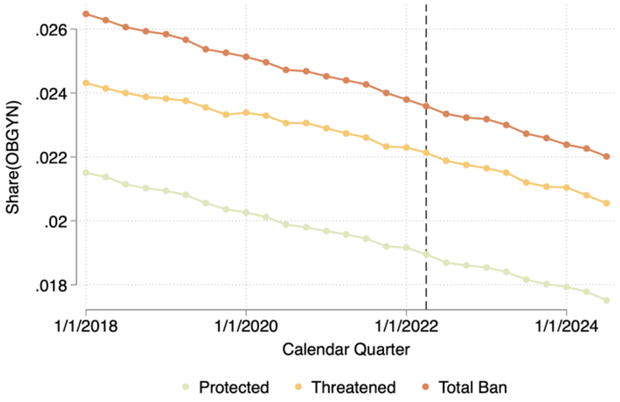

(b) Share of all physicians who are OBGYNs and men

© 2025 Staiger B et al. *JAMA Network Open*.

eFigure 4. Trends in the Percent Change in OBGYNs, Pediatricians, Internists, and Anesthesiologists in Total Ban States

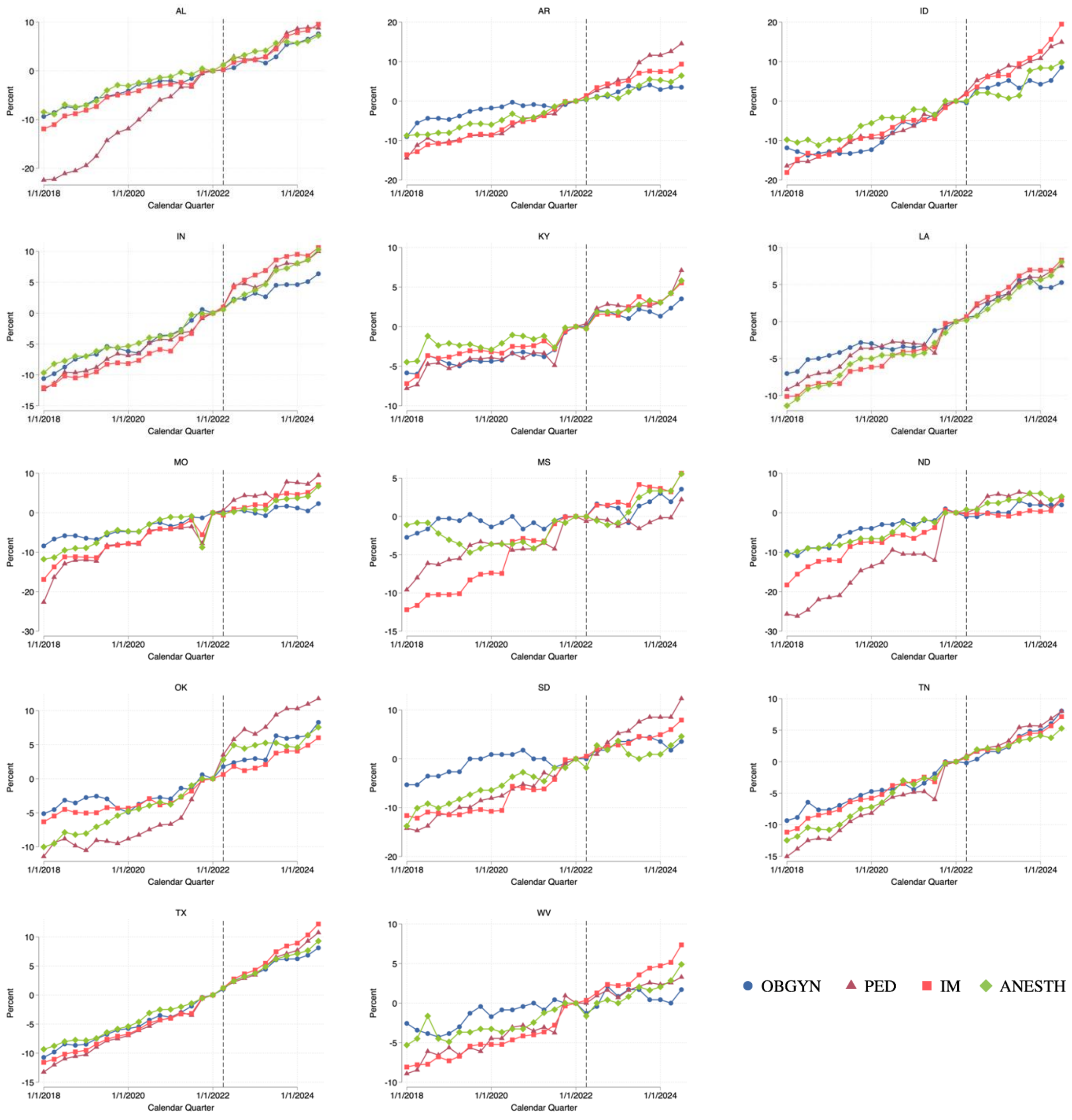

Note: Pediatrics is abbreviated by "PED," internal medicine is abbreviated by "IM," and anesthesiology is abbreviated by "ANESTH." Percent change is calculated separately for each specialty relative to the number of physicians in that specialty in a given state in 2022Q1.

© 2025 Staiger B et al. *JAMA Network Open*.

eFigure 5. Trends in State Licenses Associated With OBGYNs, 2018 – 2024

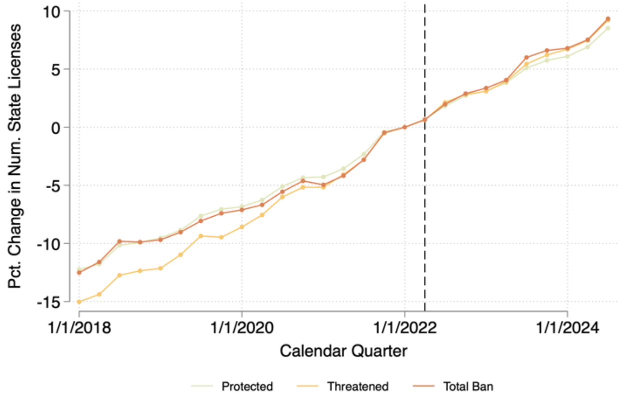

(a) Percent change in number of state licenses associated with OBGYNs per policy environment

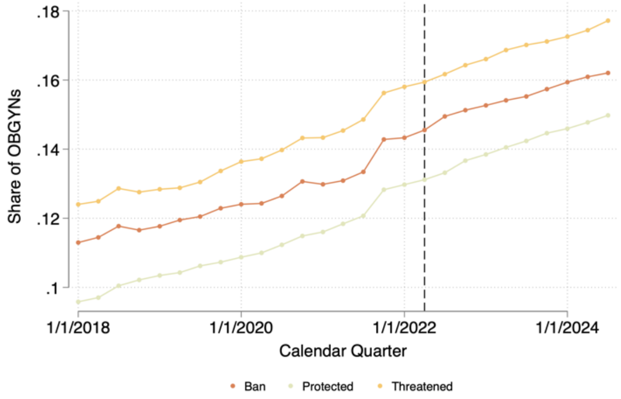

(b) Share of OBGYNs with >1 state license

Note: Percent change is calculated relative to the number of state licenses associated with OBGYNs in a given policy environment in 2022Q1.

© 2025 Staiger B et al. *JAMA Network Open*.

eFigure 6. Trends in the Count of OBGYNs per Policy Environment, Business State Definition

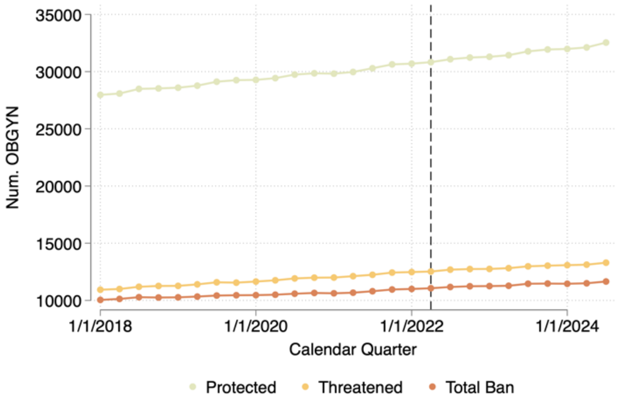

(a) Count

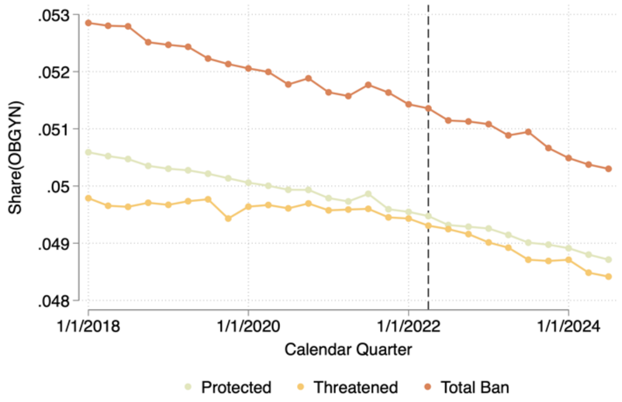

(b) Share of all physicians who are OBGYNs

Notes: The business state of an OBGYN's National Plan and Provider Enumeration System record is used instead of the practice state.

© 2025 Staiger B et al. *JAMA Network Open*.
