# Supplementary material for: Obstetrician and Gynecologist Physicians’ Practice Locations Before and After the Dobbs Decision
Source: JAMA Netw Open. 2025 Apr 21;8(4):e251608. doi: 10.1001/jamanetworkopen.2025.1608 (PMC12013358; doi:10.1001/jamanetworkopen.2025.1608)
Supplement: Supplement 2. — Data Sharing Statement [file jamanetwopen-e251608-s002.pdf]

Data Sharing Statement

Staiger. Obstetrician and Gynecologist Physicians' Practice Locations Before and After the Dobbs Decision. *JAMA Netw Open*. Published April 04, 2025.
doi:10.1001/jamanetworkopen.2025.1608

Data
Data available: Yes
Data types: Data (not involving human participants)
How to access data: This study uses monthly information on clinicians that have a national provider identifier (NPI). Up-to-date monthly data can be obtained online: https://download.cms.gov/nppes/NPI_Files.html. The authors will upload historical, previously-downloaded monthly data as part of their replication package.
When available: With publication

Supporting Documents
Document types: Statistical/analytic code
How to access documents: A complete replication package with code and publicly available data will be posted on open ICPSR (https://www.openicpsr.org/openicpsr/) as well as on the author's github page (URL will be provided at creation of page, with publication).
When available: With publication

Additional Information
Who can access the data: Data will be made available to the public.
Types of analyses: All code and data will be made available for any type of analysis.
Mechanisms of data availability: The data and code will be hosted online in a publicly available forum.
Any additional restrictions: One dataset (IQVIA OneKey) is accessible to the authors only via a DUA and will not be provided in a public forum. This will restrict parts of the analyses that can be replicated.
